# Impact of media coverage on the drinking dynamics in the scale-free network

**DOI:** 10.1186/s40064-016-1790-8

**Published:** 2016-02-27

**Authors:** Hai-Feng Huo, Yan-Yan Wang

**Affiliations:** Department of Applied Mathematics, Lanzhou University of Technology, Lanzhou, 730050 Gansu People’s Republic of China

**Keywords:** Binge drinking, Scale-free network, Media coverage, Equilibrium, Stability

## Abstract

A binge drinking model with the impact of media in the scale-free network is proposed. The basic reproduction number $R_0$ is derived by the next generation matrix method. Stability of the alcohol free equilibrium is proved by the comparison theorem. Existence and uniqueness of the alcohol present equilibrium is also obtained. Furthermore, the permanence of the system and the influence of media coverage on the drinking dynamics are studied, and the difference between our model on heterogeneous and homogeneous networks is also discussed. Numerical simulations are presented to illustrate our theoretical results. Our results show that media coverage does not change the value of $R_{0}$ but it is an effective measure in reducing alcohol problems.

## Background

It is well known that excessive drinking is not only harm to personal health, but also induce serious consequences for the family and society. It harms the nervous system and the internal organs, furthermore, it causes cancer easily. According to the third national cancer survey in America, the consisting alcohol abuse is an important reason for the mouth, throat and esophagus cancer. The growing excessive drinking increases the risk of breast cancer by 13 % every year. In addition, there are data show that, on average, heavy drinkers die 10–20 years earlier than nondrinkers. 40 and 32 % of family divorce are caused by alcohol, in America and China, respectively (Inoue 2013; Rehm 2011; Ribes et al. 2008). At the same time, alcohol abusers often appear the phenomenon of early death or sudden suicide (Mash et al. 2014).

Heavy drinking has been treated as a chronic disease. More and more researchers study the harmfulness of binge drinking through establishing mathematical models to find some way to control the drinking behavior (Mulone 2012; Walters et al. 2013; Santonja et al. 2010; Mubayi et al. 2010; Manthey et al. 2008) and references cited therein. Mushayabasa and Bhunu (2011) established a deterministic model to assess the impact of binge drinking on the incidence of gonorrhea. Wang et al. (2014) proposed an objective functional which considered not only alcohol quitting effects but also the cost of controlling alcohol, and they investigated optimal control strategies in an alcoholism model with the help of Pontryagins Maximum Principle. Huo and Song (2012) investigated a more realistic binge drinking model with two stages, in which the youths with alcohol problems were divided into those who admitted the problem and those who did not admit it.

Media, being the prime source of information, can not only influence the individuals’ behavior, but also affect the governments’ attitude. Generally, an effective method of reducing the spread of the disease is to make people understand the preventive measures as soon as possible. And media coverage has played a good role in controlling the infectious diseases. Many scholars introduced mathematical models with the impact of media to reduce the contact rate (Misra et al. 2011; Pang and Cui 2009; Samanta et al. 2013) and references cited therein. Liu and Cui (2008) improved a general SIR model by using the type II functional response function to depict the transmission rate decreased by media coverage, they found that effective media measures can reduce the number of infections. Huo and Wang (2014) developed a nonlinear binge drinking model with the effect of awareness programs, they assumed that the cumulative density of media increased at a rate proportional to the number of heavy drinkers. Their results showed that media coverage was an effective method in reducing alcohol problems.

However, the above mentioned alcohol models are all based on the assumption of homogeneous mixing, they ignore the influence of alcohol propagation space. In fact, the contact process of population can not be uniform collision, different people contact person may be entirely different in per unit of time. Then studying the drinking dynamics in the networks is more realistically.

With the rise of scientific researches on the network, the importance of heterogeneous social networks is perceived gradually. The complex network consists of a large number of nodes and edges, in which each node represents an individual in the real system, and each edge between two nodes represents the relationship between individuals. If a node has *k* edges, we define the node’s degree is *k*. A basic topological property in the networks is the degree distribution *p*(*k*), which is defined as the probability that a vertex randomly chosen has *k* links (Newman 2003). In general, we often study models in the scale-free network, since it considers two important characteristics of the real-world network: growth and connection tendency. Many networks such as the social network, the internet and the World Wide Web have been found to be scale-free networks, it means that the degree distribution follows a power law $p(k)\sim c k^{-\gamma }$, with $2<\gamma \le 3$, where *c* satisfies the equality of $\sum \nolimits_{k = 1}^n {p(k)} = 1$ (Liu et al. 2013).

Infectious diseases models in complex networks have been studied extensively, Shang (2013) studied a discrete-time SIS epidemic process in random networks. Local awareness, global awareness and contact awareness, were considered. Based on the stability theory of matrix difference equation, they derived analytically the epidemic threshold and found that both local and contact awareness can raise the epidemic threshold, while the global awareness only decreases the epidemic prevalence. Using a modified SIS (susceptible–infected–susceptible) model, Shang (2013) investigated the effects of three forms of awareness (i.e., contact, local, and global) on the spread of a disease in a random network. Connectivity-correlated transmission rates were assumed. By using the mean-field theory and numerical simulation, they showed that both local and contact awareness can raise the epidemic thresholds while the global awareness cannot. Their results showed that individual behaviors in the presence of an infectious disease had a great influence on the epidemic dynamics. Shang (2015) addressed two forms of individual awareness (i.e., the risk perception of an emerging epidemic). Contact awareness that increased with individual contact number, and local awareness that increased with the fraction of infected contacts. By extending the probability generating functionology, they showed that it was possible to track the evolution of the degree distributions among susceptible and infected individuals when the underlying network of contacts was represented by a semi-random configuration model. For the other infectious diseases models in the networks, we referred to Jin et al. (2011), Zhang and Jin (2011), Wang et al. (2012), Li et al. (2014), Wang and Jin (2013), Wang et al. (2013) and references cited therein.

Motivated by the above, in this paper, we construct a binge drinking model with the influence of media coverage in the scale-free network, which ignores the recruitment and death. Due to the effect of media coverage, nondrinkers form a separate class *X*(*t*) of those who are aware of risk and avoid contacting with the heavy drinkers.

The organization of this paper is as follows: In “The model formulation” section, we propose a binge drinking model with the influence of media coverage in the scale-free network. In “Analysis of the model” section, we prove the stability of the alcohol free equilibrium, the uniqueness of the alcohol present equilibrium and the permanence of the system. In “Numerical simulation” section, we present some numerical simulations. Some summaries and discussions are given in last section.

## The model formulation

### System description

To account for the heterogeneity in the contacts amongst individuals, we divide the total population *N* into *n* (*n* is the maximum degree) groups according to the degree of nodes, that is to say,

1 $$N=N_{1}+N_{2}+\cdots +N_{n},$$

then the degree distribution

2 $$p(k)=\frac{N_{k}}{N}.$$

The value

3 $$\langle k\rangle =\sum _{k=1}^{n} kp(k),$$

is the mean degree. We further divide the groups into three classes according to the alcohol consumption: $S_{k}(t)$, $X_{k}(t)$, $I_{k}(t)$. $S_{k}(t)$ represents the number of nondrinkers or moderate drinkers whose degree is *k*; $X_{k}(t)$ represents the number of the aware population with degree *k*, who avoids contacting with binge drinkers; $I_{k}(t)$ represents the number of binge drinkers with degree *k*; *M*(*t*) represents the cumulative density of awareness programs driven by media. d’Onofrio et al. (2007) considered two distinct possibilities: (a) *M*(*t*) only summarizes information about the current state of the disease, i.e. *M*(*t*) only depends on current values of state variables, and (b) *M*(*t*) also summarizes information about past values of state variables. Motivated by Wang et al. (2013), we assume that *M*(*t*) only depends on current values of state variables and is a linear function of the current prevalence of the binge drinkers. Then

4 $$N_{k}(t)=S_{k}(t)+X_{k}(t)+I_{k}(t),\quad k=1,2,\ldots ,n.$$

**Fig. 1:**
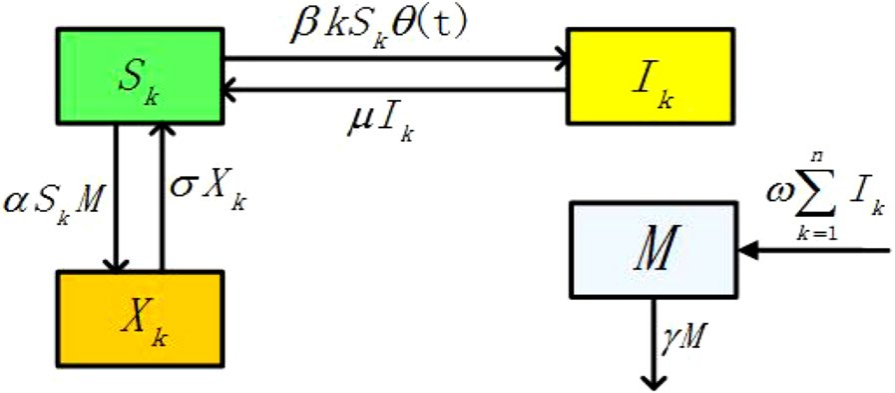
Transfer diagram of model (5)

The model’s structure is shown in Fig. 1. The transfer diagram leads to the following system of $3n+1$ ordinary differential equations

5 $$\begin{aligned} \frac{dS_{k}(t)}{dt} &= -\beta k S_{k} \theta (t)-\alpha S_{k} M + \mu I_{k}+\sigma X_{k},\\ \frac{dX_{k}(t)}{dt} &= \alpha S_{k} M-\sigma X_{k}, \nonumber \\ \frac{dI_{k}(t)}{dt} &= \beta k S_{k} \theta (t)-\mu I_{k}, \quad k=1,2,\ldots ,n,\\ \frac{dM(t)}{dt} &= \omega \sum ^{n}_{k=1} I_{k}-\gamma M. \end{aligned}$$

where $\theta (t) = \frac{1}{\langle k\rangle }\sum ^{n}_{k=1} kp(k) \frac{I_{k}}{ N_{k}}$ represents the probability that an edge of nondrinkers or moderate drinkers links to binge drinkers in uncorrelated networks, and it is between 0 and 1. We let the transmission coefficient be $\beta$. Then the number of $S_{k}$, who has *k* links, turning to $I_{k}$ is $k \beta S_{k} \theta (t)$. For simplicity, in our model, we only consider the impact of media on nondrinkers or moderate drinkers, then $S_{k}$ form a new compartment $X_{k}$, in which people avoid contacting with binge drinkers. When binge drinkers receive treatments or other interventions, they will recover to $S_{k}$ at rate $\mu$. This method has been used in literature (Misra et al. 2011; Huo and Wang 2014). $\alpha$ represents the dissemination rate of awareness among nondrinkers or moderate drinkers. Since the influence of media can not be permanent, when the awareness of avoiding contacting with alcoholics is gradually fading, $X_{k}$ will no longer consciously cut off the contact with alcoholics. Then they will return to $S_{k}$ at the transformation rate $\sigma$. $\omega$ is a positive constant, and represents the growth rate of media. $\gamma$ represents the depletion rate of media coverage resulted by ineffective measures. All the parameters can be found in Table 1, and they are positive constants.

**Table 1:** The parameters description of model (5)

Parameter	Description
$\beta$	The transmission coefficient for nondrinkers or moderate drinkers turning to binge drinkers
$\mu$	The recovery rate of binge drinkers
$\alpha$	The dissemination rate of awareness among nondrinkers or moderate drinkers
$\sigma$	The transformation rate from aware individuals to nondrinkers or moderate drinkers
$\omega$	The growth rate of media
$\gamma$	The depletion rate of media resulted by ineffective measures

According to the practical significance of system (5), if binge drinkers extinct, there is no meaning to report. It means that binge drinkers are the source of media coverage in our model. Thus we assume that the density of media coverage is equal to zero when the number of binge drinkers is equal to zero. In addition, nondrinkers or moderate drinkers $S_{k}$ is the unique source of the aware population $X_{k}(t)$, so $X_{k}(t)$ is also equal to zero when there is no media coverage.

### Positivity and boundedness of solutions

#### **Lemma 1**

*Let*$(S_{1}(t),\;X_{1}(t),\;I_{1}(t),\;\ldots , \;S_{n}(t),\;X_{n}(t),\;I_{n}(t),\;M(t))$*be the solution of system* (5), if $S_{k}(0)>0,\;X_{k}(0)>0,\;I_{k}(0)>0$, $M(0)>0$*and*$\theta (0)>0$, *then for*$k=1,2,\ldots ,n$, *then*$S_{k}(t)>0,X_{k}(t)>0,I_{k}(t)>0$, $M(t)>0$*and*$\theta (t)>0$*for all*$t>0$.

#### *Proof*

Adding the first three equations of system (5), we have

$$\frac{dN_{k}(t)}{dt}=\frac{dS_{k}(t)}{dt}+\frac{dX_{k}(t)}{dt}+\frac{ dI_{k}(t)}{dt}=0.$$

Substituting the third equation of system (5) into the formula of $\theta (t)$, we get

$$\begin{aligned} \theta ^{\prime }(t) &= \frac{1}{{\left\langle k \right\rangle }} \sum \limits _{k = 1}^n {kp(k)\frac{{{{I^{\prime }}_k}(t){N_k}(t) - 0}}{{ N_k^2(t)}}} = \frac{1}{{\left\langle k \right\rangle }}\sum \limits _{k = 1}^n {kp(k)\frac{{{{I^{\prime }}_k}(t)}}{{{N_k}(t)}}} \\& = \theta (t) \left[ \frac{1}{{ \left\langle k \right\rangle }}\sum \limits _{k = 1}^n {kp(k)\frac{{\beta k{S_k }(t)}}{{{N_k}(t)}}} - \mu \right] , \end{aligned}$$

it implies that

$$\theta (t) = \theta (0)exp \left[ - \mu t + \frac{1}{{\left\langle k \right\rangle }}\int _0^t {\sum \limits _{k = 1}^n {kp(k)\frac{{\beta k{S_k}(\tau )}}{{{N_k} (\tau )}}} d\tau } \right].$$

Since $\theta (0)>0$, we obtain $\theta (t)>0$ for all $t>0$. Using the continuity of $S_{k}(t)$, since $S_{k}(0)>0$, we can find a small $\delta >0$ , such that $S_{k}(t)>0$ for $0<t<\delta$. Now we prove that $S_{k}(t)>0$ for all $t>0$. If not, we assume a contradiction that there exists $t_{1}\ge \delta >0$, such that $S_{k}(t_{1})=0$ and $S_{k}(t)>0$ for all $0<t<t_{1}$. From the third equation of system (5), we have

$$I_{k}^{\prime }(t)+\mu I_{k}(t)=\beta kS_{k}(t)\theta (t)> 0, \quad 0<t<t_{1},$$

then

$$I_{k}(t)> I_{k}(0)e^{-\mu t}>0, \quad 0<t<t_{1}.$$

From the last equation of system (5), we have

$$M^{\prime }(t) > 0 - \gamma M(t), \quad 0 < t < {t_1},$$

it follows that

$$M(t) > M(0){e^{ - \gamma t}} > 0, \quad 0 < t < {t_1}.$$

Similarly, from the second equation of system (5), we have

$$X_{k}^{\prime }(t)+\sigma X_{k}(t)=\alpha S_{k}(t)M(t)>0, \quad 0<t<t_{1},$$

then

$$X_{k}(t)> X_{k}(0)e^{-\sigma t}> 0, \quad 0<t<t_{1}.$$

Using the continuity of $X_{k}(t)$ and $I_{k}(t)$, we have $X_{k}(t_{1})\ge 0$ and $I_{k}(t_{1})\ge 0$, so

$$S_{k}^{\prime }(t_{1})=\mu I_{k}(t_{1})+\sigma X_{k}(t_{1})\ge 0.$$

That is to say, $S_{k}(t)\le 0$ for $0<t<t_{1}$, which is contradictory. Thus $S_{k}(t)>0$ for all $t>0$. Similarly, we can prove that $X_{k}(t)>0$, $I_{k}(t)>0$ and $M(t)>0$ for all $t>0$. Hence the proof is completed. $\square$

#### **Lemma 2**

*All feasible solutions of system* (5) *are in the following bounded region*

6 $$\begin{aligned} \Omega &= \left\{ (S_{1}(t),\;X_{1}(t),\;I_{1}(t),\;\ldots ,\;S_{n}(t), \;X_{n}(t),\;I_{n}(t), \;M(t)) \in {R_ +^{3n + 1}}\left| {0 \le {S_k}(t),{X_k}(t),}\right. \right.\\&\quad \left. {I_k}(t) \le {N_k}(t),{S_k}(t) + {X_k}(t) + {I_k}(t) = {N_k}(t),1 \le k \le n,0 \le M(t) \le \frac{{\omega N}}{\gamma } \right\} . \end{aligned}$$

#### *Proof*

Since ${{d{N_k}(t)}/{dt}} = 0$, then $S_{k}(t) + X_{k}(t) + I_{k}(t)=N_{k}(t)$ is constant. Furthermore, the positivity of solutions is proved in Lemma 1, it implies that $0 \le {S_k} (t),{X_k}(t),{I_k}(t) \le {N_k}(t)$. From the last equation of system (5), we have

$$0-\gamma M\le M^{\prime }(t)\le \omega N-\gamma M,$$

it follows that

$$0\le M(0)e^{-\gamma t}\le M(t)\le \frac{\omega N}{\gamma }+M(0)e^{-\gamma t},$$

thus

$$\mathop {\lim }\limits _{t \rightarrow \infty } \sup M(t) \le \frac{{\omega N}}{ \gamma }.$$

So the region $\Omega$ is a positively invariant set of system (5). This completes the proof of Lemma 2. $\square$

## Analysis of the model

### The basic reproduction number and alcohol free equilibrium

We define the relative density of nondrinkers or moderate drinkers, aware population and binge drinkers with degree *k* by $s_{k}(t)=\frac{S_{k}(t)}{ N_{k}},\ x_{k}(t)=\frac{X_{k}(t)}{N_{k}},\ i_{k}(t)=\frac{I_{k}(t)}{N_{k}},$ then $s_{k}(t)+x_{k}(t)+i_{k}(t)=1$. So system (5) can be written as

7 $$\begin{aligned} \frac{dx_{k}(t)}{dt} &= \alpha (1-x_{k}-i_{k}) M-\sigma x_{k}, \\ \frac{di_{k}(t)}{dt} &= \beta k (1-x_{k}-i_{k}) \theta (t)-\mu i_{k},\quad k=1,2,\ldots ,n, \\ \frac{dM(t)}{dt} &= \omega N \sum ^{n}_{k=1}p(k) i_{k}-\gamma M,\end{aligned}$$

where $$\theta (t)=\frac{1}{\langle k\rangle }\sum ^{n}_{k=1} kp(k) i_{k}$$. Let

8 $$\begin{aligned} \Gamma &= \left\{ ({x_1}(t),{i_1}(t),\ldots ,{x_n}(t),{i_n}(t),M(t)) \in {R_+^{2n + 1}}\left| {0 \le {x_k}(t),{i_k}(t) \le 1,}\right. \right. \nonumber \\&\quad \left. 0 \le {x_k}(t) + {i_k}(t) \le 1,1 \le k \le n,0 \le M(t) \le \frac{{\omega N} }{\gamma } \right\} . \end{aligned}$$

It can be verified that region $\Gamma$ is a positively invariant set of system (7).

System (7) has a unique alcohol free equilibrium given by

9 $$E_{0}=(0, 0, \ldots, 0, 0, \ldots, 0, 0, 0).$$

Using the next generation matrix method (Driessche and Watmough 2002), we calculate the basic reproduction number $R_{0}=\rho (FV^{-1})$. In our case, the production of new binge drinkers ${\mathscr {F}}$ and the rate of transfer of individuals ${\mathscr {V}}$ are given by

$$\begin{aligned} {\mathscr {F}} = {\left( {\begin{array}{c} {\beta (1 - {x_1} - {i_1})\theta }\\ {\beta 2 (1 - {x_2} - {i_2})\theta }\\ \vdots \\ {\beta n (1 - {x_n} - {i_n})\theta }\\ 0\\ 0\\ \vdots \\ 0\\ 0 \end{array}} \right) _{2n + 1}},\quad {\mathscr {V}} = {\left( {\begin{array}{c} {\mu {i_1}}\\ {\mu {i_2}}\\ \vdots \\ {\mu {i_n}}\\ {\sigma {x_1} - \alpha (1 - {x_1} - {i_1})M}\\ {\sigma {x_2} - \alpha (1 - {x_2} - {i_2})M}\\ \vdots \\ {\sigma {x_n} - \alpha (1 - {x_n} - {i_n})M}\\ {\gamma M - \omega N\sum \limits _{k = 1}^n {p(k){i_k}} } \end{array}} \right) _{2n + 1}}. \end{aligned}$$

Calculating the Jacobian matrices of ${\mathscr {F}}$ and ${\mathscr {V}}$ at $E_{0}$ as follows

$$\begin{aligned} F & = D{\mathscr {F}}(E_{0}) ={\left( {\begin{array}{ccc} {{F_{11}}}&\quad 0&\quad 0\\ 0&\quad 0&\quad 0\\ 0&\quad 0&\quad 0 \end{array}} \right) _{(2n+1) \times (2n+1)}}, \\ V &= D{\mathscr {V}}(E_{0}) ={ \left( {\begin{array}{ccc} {{V_{11}}}&\quad 0&\quad 0\\ 0&\quad {{V_{22}}}&\quad {{V_{23}}}\\ {{V_{31}}}&\quad 0&\quad {{V_{33}}} \end{array}} \right)_{(2n+1) \times (2n+1)}}, \end{aligned}$$

where

$$\begin{aligned} {F_{11}} &= \frac{\beta }{{\langle k\rangle }}\left( {\begin{array}{cccc} {p(1)}&\quad {2p(2)}&\quad \cdots &\quad {np(n)}\\ {2p(1)}&\quad {{2^2}p(2)}& \quad \cdots & \quad {2np(n)}\\ \vdots & \quad \vdots & \quad \ddots & \quad \vdots \\ {np(1)}& {n2p(2)}& \cdots & {{n^2}p(n)} \end{array}} \right) _{n \times n}, \\ {V_{23}} &= \left( {\begin{array}{cccc} { -\alpha }&\quad { -\alpha }&\quad \cdots&\quad { -\alpha } \end{array}} \right) _{n}^\mathrm{T}, \\ {V_{31}}&= {\left( {\begin{array}{cccc} {-\omega Np(1)}&\quad { -\omega Np(2)}&\quad \cdots&\quad { -\omega Np(n)} \end{array}} \right) _{n}}, \end{aligned}$$

$V_{11} = \mu E, V_{22} = \sigma E, V_{33} = \gamma$, *E* represents a unit matrix and 0 represents a zero matrix. Finally, we calculate the basic reproduction number $R_{0}=\frac{\beta \langle k^{2}\rangle }{\mu \langle k\rangle }$.

Following Theorem 2 of Driessche and Watmough (2002), we have the following result on the local stability of $E_{0}$.

#### **Theorem 1**

*The alcohol free equilibrium*$E_{0}$*of system* (7) *is locally asymptotically stable if*$R_{0}<1$, *but unstable if*$R_{0}>1$.

Next, we will prove the globally asymptotically stability of $E_{0}$.

#### **Theorem 2**

*The alcohol free equilibrium*$E_{0}$*of system* (7) *is globally asymptotically stable if*$R_{0}<1$.

#### *Proof*

Let ${i_1} = {y_1},{i_2} = {y_2}, \ldots ,{i_n} = {y_n},{x_1} = {y_{n + 1}},{x_2} = {y_{n + 2}}, \ldots ,{x_n} = {y_{2n}},M = {y_{2n + 1}},y = {({y_1},{y_2}, \ldots ,{y_{2n + 1}})^T}$, $g(j) = \frac{{jp(j)}}{{\left\langle k \right\rangle }}$, then system (7) can be written in terms of

10 $$\frac{{dy}}{{dt}} = Ay + N(y),$$

where

$$\begin{aligned} A &= \left( {\begin{array}{ccc} {{A_{11}}}&{}\quad {{0}}&{}\quad {{0}}\\ {{0}}&{}\quad {{A_{22}}}&\quad {{A_{23}}}\\ {{A_{31}}}&\quad {{0}}&\quad {{A_{33}}}\\ \end{array}} \right) , \\ {A_{11}} &= {\left( {\begin{array}{cccc} {\beta g(1) - \mu }&{}\quad {\beta g(2)}&\quad \cdots &\quad {\beta g(n)}\\ {\beta 2g(1)}& \quad {\beta 2g(2) - \mu }& \quad \cdots & \quad {\beta 2g(n)}\\ \vdots & \quad \vdots & \quad \ddots & \quad \vdots \\ {\beta ng(1)}& \quad {\beta ng(2)}& \quad \cdots & \quad {\beta ng(n) - \mu } \end{array}} \right) _{n \times n}}, \\ {A_{23}} &= \left( {\begin{array}{cccc} { \alpha }&\quad { \alpha }&\quad \cdots&\quad { \alpha } \end{array}} \right) _{n}^\mathrm{T}, \\ {A_{31}} &= {\left( {\begin{array}{cccc} {\omega Np(1)}&\quad {\omega Np(2)}&\quad \cdots&\quad {\omega Np(n)} \end{array}} \right) _{n}}, \\ {A_{22}} &= - \sigma E,{A_{33}} = - \gamma , \end{aligned}$$

and

$$\begin{aligned} N(y) = - {\left( {\begin{array}{c} {\beta \theta ({y_1} + {y_{n + 1}})}\\ {2\beta \theta ({y_2} + {y_{n + 2}})}\\ \vdots \\ {n\beta \theta ({y_n} + {y_{2n}})}\\ {\alpha {y_{2n+1}}({y_1} + {y_{n + 1}})}\\ {\alpha {y_{2n+1}}({y_2} + {y_{n + 2}})}\\ \vdots \\ {\alpha {y_{2n+1}}({y_n} + {y_{2n}})}\\ 0 \end{array}} \right) _{2n + 1}}. \end{aligned}$$

Thus

11 $$\frac{{dy}}{{dt}} \le Ay.$$

Considering the following linear system

12 $$\frac{{dy}}{{dt}} = Ay.$$

If $R_{0} < 1$, all eigenvalues of *A* have negative real parts (Driessche and Watmough 2002). It follows that system (12) is stable whenever $R_{0} < 1$. So ${i_k}(t) \rightarrow 0, {x_k}(t) \rightarrow 0, M(t) \rightarrow 0$, as $t \rightarrow \infty$, for this linear system. Since (12) is a quasi monotone system, by the comparison theorem (Lakshmikantham et al. 1989), the nonlinear system (7) follows that ${i_k}(t) \rightarrow 0,{x_k}(t) \rightarrow 0,M(t) \rightarrow 0,$ as $t \rightarrow \infty ,$ for $R_{0} < 1.$ So the alcohol free equilibrium $E_{0}$ of system (7) is globally asymptotically stable. The proof is complete. $\square$

### The existence and uniqueness of the alcohol present equilibrium

#### **Theorem 3**

*If*$R_{0}>1$, *system* (7) *has a unique alcohol present equilibrium*

$${E^*}(x_1^*,i_1^*,\ldots ,x_n^*,i_n^*,{M^*}).$$

#### *Proof*

Let right side of system (7) be equal to 0, we obtain the following system

13 $$\begin{aligned} \alpha (1 - x_k^* - i_k^*){M^*} - \sigma x_k^* &= 0,\\ \beta k(1 - x_k^* - i_k^*)\theta - \mu i_k^* &= 0,\\ \omega N{i^*} - \gamma {M^*} &= 0. \end{aligned}$$

where ${i^*} = \sum \nolimits _{k = 1}^n {p(k)i_k^*} > 0.$ From the third equation of system (13), we have

14 $${M^*} = \frac{{\omega N{i^*}}}{\gamma }.$$

Substituting (14) into the first equation of system (13), we have

15 $$x_k^* = \frac{{\alpha \omega N{i^*} - \alpha \omega N{i^*}i_k^*}}{{\alpha \omega N{i^*} + \gamma \sigma }}.$$

Substituting (15) into the second equation of system (13), we obtain

16 $$\alpha \mu \omega N{i^*}i_k^* -\gamma \sigma \beta k \theta + \gamma \sigma \beta k \theta i_k^* + \gamma \sigma \mu i_k^* = 0.$$

Multiplying Eq. (16) by *p*(*k*) and summing over *k*, we obtain the result

17 $$\alpha \mu \omega N{({i^*})^2} + \gamma \sigma \mu {i^*} - \gamma \sigma \beta \langle k\rangle (\theta - {\theta ^2}) = 0.$$

According to the definition of $i^{*}$, we have

18 $${i^*} = \frac{{\ - \gamma \sigma \mu + \sqrt{\Delta }}}{{2\alpha \mu \omega N} },$$

where $\Delta = {(\gamma \sigma \mu )^2} + 4\alpha \mu \omega N\gamma \sigma \beta \langle k\rangle \theta (1 - \theta ),\theta \in [0,1].$ On the other hand, we get the following equation from (16) is that

19 $$i_k^* = \frac{{\gamma \sigma \beta k\theta }}{{\alpha \mu \omega N{i^*} + \gamma \sigma \beta k\theta + \gamma \sigma \mu }}.$$

Substituting (18) into (19), we have

20 $$i_k^* = \frac{{2\gamma \sigma \beta k\theta }}{{\gamma \sigma \mu + 2\gamma \sigma \beta k\theta + \sqrt{\Delta }}}.$$

Substituting (20) into the expression of $\theta (t) = \frac{1}{{\langle k\rangle }}\sum \nolimits _{k = 1}^n {kp(k)i_k^*} ,$ then we obtain a self-consistency equation as follows

21 $$\theta = \frac{1}{{\langle k\rangle }}\sum \limits _{k = 1}^n {kp(k)\frac{{ 2\gamma \sigma \beta k\theta }}{{\gamma \sigma \mu + 2\gamma \sigma \beta k\theta + \sqrt{\Delta }}}} .$$

If we let

22 $$f(\theta ) = 1 - \frac{2}{{\langle k\rangle }}\sum \limits _{k = 1}^n {kp(k) \frac{{\gamma \sigma \beta k}}{{\gamma \sigma \mu + 2\gamma \sigma \beta k\theta + \sqrt{\Delta }}}} ,$$

then Eq. (21) is equivalent to the following equation

23 $$\theta f(\theta ) = 0.$$

Obviously, Eq. (23) has a trivial solution $\theta =0.$ For

24 $$f^{\prime }(\theta ) = \frac{2}{{\langle k\rangle }}\sum \limits _{k = 1}^n { kp(k)\frac{A}{{B^2}}},$$

where

$$\begin{aligned} A &= 2{\gamma ^2}{\sigma ^2}{\beta ^2}k[k + \alpha \mu \omega N\langle k\rangle (1 - 2\theta )/\sqrt{\Delta }], \\ B &= \gamma \sigma \mu + 2\gamma \sigma \beta k\theta + \sqrt{\Delta }> 0, \end{aligned}$$

hence, $f^{\prime }(0) > 0.$ Through a similar derivation, we obtain that

25 $$f^{\prime \prime }(\theta ) = \frac{2}{{\langle k\rangle }}\sum \limits _{k = 1}^n {kp(k)} \frac{{{B^2}\frac{{dA}}{{d\theta }} - 2AB\frac{{dB}}{{d\theta }} }}{{B^4}},$$

where

$$\begin{aligned} \frac{{dA}}{{d\theta }} &= \frac{{2{\gamma ^2}{\sigma ^2}{\beta ^2} k \left[ - 2\alpha \mu \omega N\langle k\rangle \sqrt{\Delta }- 2{\alpha ^2}{\mu ^2}{\omega ^2}{ N^2}\gamma \sigma \beta {{\langle k\rangle }^2}{{(1 - 2\theta )}^2}/\sqrt{\Delta }\right] }}{\Delta } < 0, \\ \frac{{dB}}{{d\theta }} &= 2\gamma \sigma \beta \left[ k + \frac{{\alpha \mu \omega N\langle k\rangle (1 - 2\theta )}}{{\sqrt{\Delta }}}\right] , \end{aligned}$$

then

$$\begin{aligned} A\frac{{dB}}{{d\theta }} &= 4{\gamma ^3}{\sigma ^3}{\beta ^3}k{ \left[ k + \frac{{ \alpha \mu \omega N\langle k\rangle (1 - 2\theta )}}{{\sqrt{\Delta }}}\right] ^2} > 0, \\ {B^2}\frac{{dA}}{{d\theta }} - 2AB\frac{{dB}}{{d\theta }} &= B \left( B\frac{{dA}}{{ d\theta }} - 2A\frac{{dB}}{{d\theta }}\right) < 0, \end{aligned}$$

thus $f^{\prime \prime }(\theta ) < 0,$ that is to say, $f(\theta )$ is a convex function for $\theta \in [0,1].$ Furthermore, when $R_{0}>1,$

$$\begin{aligned} f(1) &= 1 - \frac{2}{{\langle k\rangle }}\sum \limits _{k = 1}^n {kp(k)\frac{{ \gamma \sigma \beta k}}{{\gamma \sigma \mu + 2\gamma \sigma \beta k + \gamma \sigma \mu }}} > 1 - \frac{2}{{\langle k\rangle }}\sum \limits _{k = 1}^n { kp(k)\frac{{\gamma \sigma \beta k}}{{2\gamma \sigma \beta k}}} = 1 - \frac{{ \langle k\rangle }}{{\langle k\rangle }} = 0, \\ f(0) &= 1 - \frac{2}{{\langle k\rangle }}\sum \limits _{k = 1}^n {kp(k)\frac{{ \gamma \sigma \beta k}}{{\gamma \sigma \mu + \gamma \sigma \mu }}} = 1 - \frac{2}{{\langle k\rangle }}\frac{{\gamma \sigma \beta \sum \limits _{k = 1}^n {{k^2}p(k)} }}{{2\gamma \sigma \mu }} = 1 - \frac{{\beta \langle {k^2} \rangle }}{{\mu \langle k\rangle }} = 1 - {R_0} < 0. \end{aligned}$$

So there exists a unique positive equilibrium ${E^*} (x_1^*,i_1^*,\ldots ,x_n^*,i_n^*,{M^*})$ of system (7). The prove is complete. $\square$

### The permanence of the system

The permanence of system (7) is proved in the following theorem. First, we present two lemmas in Lajmanovich and Yorke (1976).

#### **Lemma 3**

*Let*$A = (a_{ij})_{n \times n}$*be an irreducible matrix, if*$a_{ij}\ge 0$*for all*$i\ne j$, *then there exists an eigenvectorzofAsuch that*$z > 0$, *and the corresponding eigenvalue iss*(*A*).

The stability modulus *s*(*A*) is defined by $s(A) = \max _{i} \{Re{\lambda _i} \}$, $i = 1,2,\ldots ,n$, where $\lambda _i$ are the eigenvalues of *A*, *Re* denotes the real part.

#### **Lemma 4**

*Consider the system*

26 $$\frac{{dy}}{{dt}}=Py+N(y),$$

*wherePis an*$n\times n$*matrix andN*(*y*) *is continuously differentiable in a region*$D\in R^n$. *Assume*

1. *the compact convex set*$C{\subset} D$*is positively invariant with respect to system* (26), *and*$0\in C$;
2. $${\lim _{y\rightarrow 0}}{{\left\| {N(y)}\right\| }/{\left\| y\right\| }}=0;$$
3. *there exist*$r>0$*and a (real) eigenvectorzof*$P^T$*such that*$(z\cdot y)\ge r\left\| y\right\|$*for all*$y\in C$;
4. $(z\cdot N(y))\le 0$*for all*$y\in C$; *and*
5. $y=0$*is the largest positively invariant set for system* (26) *contained in*$H=\{y\in C\left| {(z\cdot N(y))=0}\right. \}$.

*Then either*$y=0$*is globally asymptotically stable inC*, *or for any*${y_0} \in C-\{0\}$*the solution*$\phi (t,y_0)$*of system* (26) *satisfies*${ \liminf _{t\rightarrow \infty }}\left\| {\phi (t,{y_0})}\right\| \ge m$, *where*$m>0$, *independent of*$y_0.$*Moreover, there exists a constant solution of system* (26), $y={y^{*}}$, ${y^{*}}\in C-\{0\}.$

#### **Theorem 4**

*If*$R_{0}>1$, *system* (7) *is permanent, i.e. there exists an*$\varepsilon >0$, *such that*

$$\begin{aligned} \mathop {\lim \inf }\limits _{t \rightarrow \infty } \{ ({x_1}(t),{i_1}(t),\ldots ,{x_n }(t),{i_n}(t),M(t))\} \ge \varepsilon , \end{aligned}$$

*where*$({x_1}(t),{i_1}(t),\ldots ,{x_n}(t),{i_n}(t),M(t))$*is any solution of system* (7), *satisfying*$\Gamma$*and*$i_{k}(0)>0$.

#### *Proof*

Considering the vectorial form (10) of system (7),

1. clearly, the compact convex set $\Gamma$ in formula (8) is a positively invariant set for system (10), and $0\in \Gamma$;
2. $$\begin{aligned} 0 &\le \mathop {\lim }\limits _{y\rightarrow 0}{{\left\| {N(y)}\right\| }/ {\left\| y\right\| }} \\ &= \mathop {\lim }\limits _{y\rightarrow 0}\frac{{\sqrt{{\beta ^2} {\theta ^2}{{({y_1}+{ y_{n+1}})}^2}+\cdots +{n^2}{\beta ^2}{\theta ^2} {{({y_n}+{y_{2n}})}^2}+{ \alpha ^2}y_{2n+1}^2{{({y_1} + {y_{n+1}})}^2}+\cdots +{\alpha ^2}y_{2n+1}^2{{({ y_n}+{y_{2n}})}^2}}}}{{\sqrt{y_1^2+y_2^2+\cdots +y_{2n+1}^2}}} \\ &\le \mathop {\lim }\limits _{y\rightarrow 0}\frac{{\sqrt{({n^2}{\beta ^2}{\theta ^2}+{ \alpha ^2}y_{2n+1}^2)\left[ {{{({y_1}+{y_{n+1}})}^2}+\cdots +{{({y_n}+{y_{2n} })}^2}}\right] }}}{{\sqrt{y_1^2+y_2^2+\cdots +y_{2n+1}^2}}} \\ &\le \mathop {\lim }\limits _{y\rightarrow 0}\frac{{\sqrt{2({n^2}{\beta ^2}{\theta ^2}+ {\alpha ^2}y_{2n+1}^2)\left[ {(y_1^2+y_{n+1}^2)+\cdots +(y_n^2+y_{2n}^2)+y_{2n+1}^2}\right] }}}{{\sqrt{y_1^2+y_2^2+\cdots +y_{2n+1}^2}}} \\ &= \mathop {\lim }\limits _{y\rightarrow 0}\sqrt{2({n^2}{\beta ^2}{\theta ^2}+{\alpha ^2}y_{2n+1}^2)} \\ &= 0, \end{aligned}$$
  thus $$\mathop {\lim }\limits _{y\rightarrow 0}{{\left\| {N(y)}\right\| }/{\left\| y\right\| }}=0;$$
3. $A_{11}^T=(a_{ij})_{n\times n}$ be an irreducible matrix, and $a_{ij}\ge 0$ for all $i\ne j$, then from Lemma 3, there exists a positive eigenvector $\tilde{z}=({z_1},{z_2},\ldots ,{z_n})$ of $A_{11}^T$, and the corresponding eigenvalue is $s(A_{11}^T)$. When $R_0>1$, $s(A_{11}^T)>0$. Let $z_{n+1}=\cdots =z_{2n+1}=0$, $z=(\tilde{z},{z_{n+1}} ,\ldots ,{z_{2n+1}})=({z_1},{z_2},\ldots ,{z_{2n+1}})$, then ${A^T} z=s(A_{11}^T)z$, i.e. *z* is the eigenvector of $A^T$ corresponding eigenvalue is $s(A_{11}^T)$. Take $r={\min _{1\le i\le n}}\{{z_i}\}>0$, then $\left( {z\cdot y}\right) \ge r\left\| y\right\|$ for all $y\in \Gamma$;
4. $(z\cdot N(y))\le 0$ for all $y\in \Gamma$, since ${z_i}\ge 0$ and ${ (N(y))_i}\le 0$, $i=1,2,\ldots ,2n+1$;
5. let $H=\{y\in \Gamma \left| {(z\cdot N(y))=0}\right. \}$. If $y\in H$, then $\sum \nolimits _{k=1}^n{{z_k}\beta \theta k({y_k}+{y_{n+k}})}=0$. Since $z_k>0$, $\beta >0$, $k>0$ and $\theta (t)=\frac{1}{\langle k\rangle }\sum _{k=1}^nkp(k)y_k$, thus $y_k$ must be equal to zero. Accroding to the assume of system (5), we get that $y=0$ is the only solution contained in *H*, thus it is the largest positively invariant set for system (10).

All the hypotheses of Lemma 4 are satisfied, then system (7) is permanent. The theorem is proved completely. $\square$

#### **Conjecture**

*If*$R_0>1,$* the alcohol present equilibrium*$E^*$* of system* (7)* is globally asymptotically stable*.

#### *Remark*

**Fig. 2:**
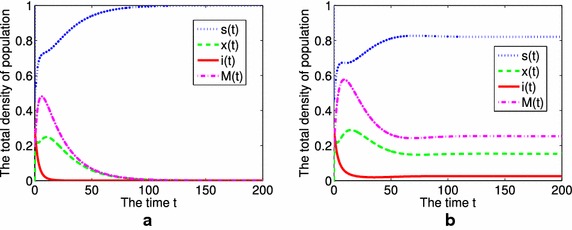
The time series for the total density of population in the scale-free network. **a** $R_{0}<1$, **b** $R_{0}>1$

We have great difficulty in proving the global stability of $E^*$. Then we only carry out simulations to test our conjecture (Figs. 2b, 4). It is still an open problem to prove the global stability of $E^*$.

## Numerical simulation

To illustrate our theoretical results, we present some numerical simulations. In the scale-free network, we take $N=1000$, $p(k) = 2{m^2}{k^{ - 3}}$ (m is the minimum degree, we take $m=3$). The values of some parameters in our model are estimated as $\mu =0.4$, $\sigma =0.2$, $\alpha =0.1$, $\beta =0.02$ or $\beta =0.04$, other parameters are cited in Wang et al. (2013) as $\gamma =0.05$, $\omega =0.0005$, $\beta =0.02$, then $R_{0}=0.7597<1$. $R_{0}= 1.5194>1$ when $\beta =0.04$.

Firstly, we investigate the time evolution for the total density of population $s(t) = \sum \nolimits_{k = 1}^n {p(k){s_k}(t)}$, $x(t) =\sum \nolimits_{k = 1}^n {p(k){x_k}(t)}$, $i(t) =\sum \nolimits_{k = 1}^n {p(k){i_k}(t)}$, and *M*(*t*). When $R_{0}<1$, ${\lim_{t \rightarrow \infty }}(x(t),i(t),M(t)) = (0,0,0)$, that is to say, the phenomenon of alcohol abuse will disappear, $E_{0}$ is globally asymptotically stable (Fig. 2a); When $R_{0}>1$, as time going on, the solution of system (7) finally tends to a unique positive constant, which means the phenomenon of alcohol abuse will be prevalent and $E^{*}$ is globally asymptotically stable (Fig. 2b).

**Fig. 3:**
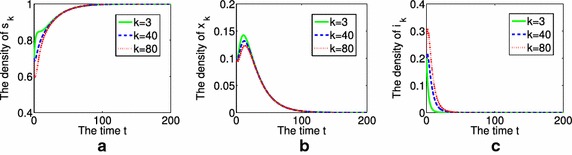
The time evolution of the density of $s_{k}$, $x_{k}$ and $i_{k}$ with different degrees $k=3$, $k=40$ and $k=80$, when $R_{0}<1$, **a** for $s_{k}$, **b** for $x_{k}$, **c** for $i_{k}$

**Fig. 4:**
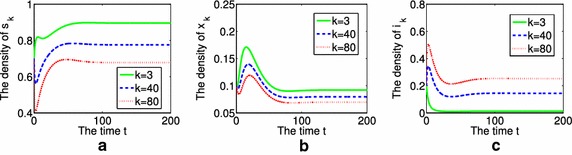
The time evolution of the density of $s_{k}$, $x_{k}$ and $i_{k}$ with different degrees $k=3$, $k=40$ and $k=80$, when $R_{0}>1$, **a** for $s_{k}$, **b** for $x_{k}$, **c** for $i_{k}$

Secondly, we present the time evolution of the density of nondrinkers or moderate drinkers $s_{k}$, aware population $x_{k}$ and binge drinkers $i_{k}$ with different degrees $k=3$, $k=40$ and $k=80$. The initial conditions are chosen as $s_{k}(0)=0.7$, $x_{k}(0)=0.1$, $i_{k}(0)=0.2$, $M(0)=0.2$. Figure 3 performs the case of $R_{0}=0.7597<1$. From Fig. 3, we know that the greater *k* is, the greater density of binge drinkers is in that group, and the slower the phenomenon of binge drinking vanishes, when $R_{0}<1$. Figure 4 performs the other case of $R_{0}= 1.5194>1$, it shows that the density of binge drinkers $i_{k}$ is proportional to the value of *k*. However, $i_{k}$ finally tends to a positive constant, the phenomenon of alcohol abuse will be prevalent.

**Fig. 5:**
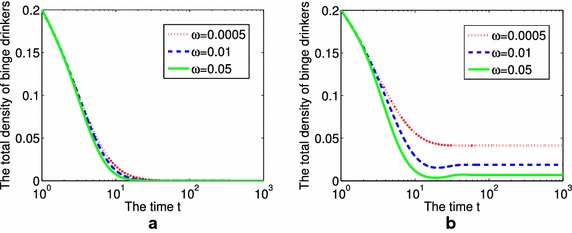
The influence of different values of $\omega$ on the total density of binge drinkers, respectively. **a** $R_{0}<1$, **b** $R_{0}>1$

Thirdly, we study the influence of parameter $\omega$ on the total density of binge drinkers (Fig. 5). With $\omega =0.0005$, 0.01, 0.05 (from top to bottom). When $R_{0}<1$, increasing the value of $\omega$ could accelerate the extinction of the phenomenon of alcohol abuse (Fig. 5a); When $R_{0}>1$, media coverage can’t change the value of $R_{0}$, but it reduces the final binge drinkers’ density largely (Fig. 5b).

## Conclusions and discussions

In this paper, we use mean-field theory to propose a binge drinking model with the impact of media in the scale-free network. The outcomes show that $E_{0}$ and $E^{*}$ are all globally asymptotically stable. It means that the phenomenon of alcohol abuse will disappear if $R_0<1$, otherwise it will be prevalent if $R_{0}>1$.

Comparing with the drinking model on homogeneous networks Huo and Wang (2014). The basic reproductive number in our model is in direct proportion to the heterogeneous parameter $\langle k^{2}\rangle \gg \langle k\rangle$. So, network heterogeneity makes drinking behavior more easily to spread. At the same time, we discuss the influence of different $\omega$ on the total density of binge drinkers, finding that media coverage is an effective measure in reducing alcohol problems though it does not change the spreading threshold.
